# Discovery of a fully ossified transverse humeral ligament during biceps tenodesis: a case report

**DOI:** 10.1016/j.xrrt.2025.08.011

**Published:** 2025-08-29

**Authors:** Hady Ezzeddine, Wendy Ghanem, Mohamad Badra, Ramzi Moucharafieh, Ali Abou Saleh, Mohammad Jomaa

**Affiliations:** a Department of Orthopaedic Surgery and Traumatology, University of Balamand, Beirut, Lebanon; b Department of Orthopedic Surgery, Clemenceau Medical Center, Beirut, Lebanon; c Department of Orthopedic Surgery, Labib Medical Center, Saida, Lebanon

**Keywords:** Transverse humeral ligament, Biceps tendon, Bicipital groove, Posttraumatic, Ossification, Osseous metaplasia

The transverse humeral ligament (THL) is a robust, *elastin*-deficient fibrous sheath that spans the intertubercular sulcus (IS) between the greater (GT) and lesser (LT) tuberosities of the humerus.^5^ Functioning as a retinaculum, it stabilizes the long head of the biceps tendon (LHBT) within the bicipital groove (BG) as the humerus glides around the relatively fixed tendon during arm elevation and rotation.^9^ Due to its lack of *elastin*, the histological identity of this fibrous structure remains debated; some investigators suggest that it represents an extension of the subscapularis tendon rather than a true ligament.^5^

Whether considered ligamentous or tendinous, ossification or calcification of the THL has not been reported in any clinical context, specifically in association with LHBT pathologies, calcific tendonitis of the rotator cuff, or calcific pathologies of other shoulder ligaments.^2^^,^^8^^,^^12^ In contrast, post-traumatic calcification and ossification of the coracoacromial and coracoclavicular ligaments are well documented.^2^

Small bone spurs measuring approximately 1 to 2 millimeters^12^ grow along the medial wall,^9^^,^^12^ lateral wall,^12^ and floor^9^ of the IS and are often encountered during shoulder arthroplasty in elderly patients. These spurs, considered degenerative in nature, appear in up to 60% of patients over 55 years of age.^12^ Their etiology is commonly attributed to chronic mechanical traction exerted by the THL under LHBT tension, analogous to degenerative enthesopathic changes observed at various tendon and ligament insertion sites throughout the body.^9^ Nevertheless, there is currently no evidence supporting a causal relationship between these spurs and chronic LHBT pathology.^12^

Although small spurs are common within the IS, complete ossification of THL resulting in osseous bridging, or “tunnelization”, of the groove and synostosis of the GT and LT remains unrecognized in the medical literature and in routine clinical practice. To date, only three isolated cadaveric specimens have demonstrated this phenomenon, each absent of clinical context.^1^^,^^3^^,^^11^

In this report, we present the first documented case of post-traumatic THL ossification in a live, nonarthritic shoulder. We provide clear chronological evidence demonstrating the absence of THL ossification at the time of injury, followed by the development of ossification several months post-trauma.

## Case report

**Figure 1:**
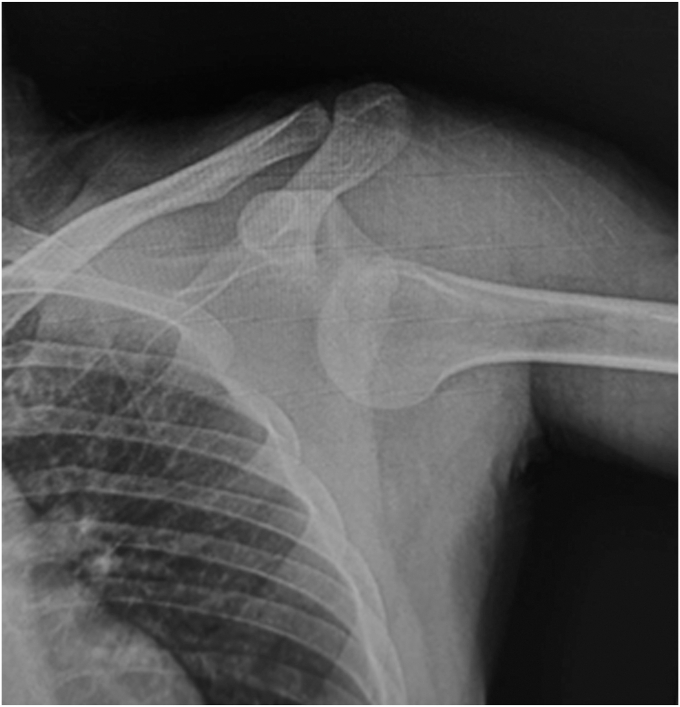
Radiograph of the left shoulder acquired on the day of polytrauma, demonstrating anteroinferior glenohumeral dislocation.

**Figure 2:**
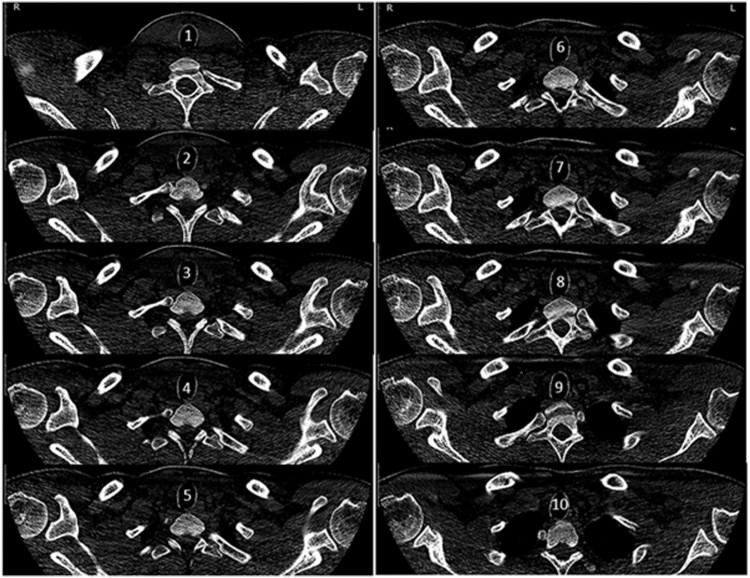
Computed tomography of the chest conducted on the day of trauma as part of standard polytrauma assessment. The image revealed no abnormal densities within or traversing the bicipital grooves bilaterally.

On August 12, 2024, a 35-year-old, right-hand dominant male commercial controller with no prior history of left shoulder pathology sustained multiple injuries in a high-speed motorcycle collision. These included a right femoral shaft fracture, right open-book pelvic fracture, and anteroinferior dislocation of the left shoulder (Fig. 1). The shoulder dislocation was promptly reduced in the emergency room, and both fractures were subsequently managed with open reduction and internal plate fixation in two different sessions. No advanced shoulder imaging was obtained at the time; however, a chest computed tomography performed on the day of trauma partially captured both shoulder girdles and confirmed the absence of abnormal densities within or spanning the BG bilaterally (Fig. 2). Conservative management of the left shoulder was initiated, and the patient underwent extensive upper and lower limb rehabilitation over the following months. During rehabilitation, the patient reported progressively worsening pain, stiffness, and weakness in the left shoulder. He did not report instability symptoms, as stiffness was more predominant. Two cortisone injections did not help alleviate his symptoms.

On March 21, 2025, magnetic resonance imaging (MRI) of the left shoulder was obtained, followed by referral to our clinic. On physical examination, the patient actively reached 120° of forward elevation and 100° of lateral elevation (vs. 155° passively). External rotation at the side (ER1) was 35° (45° passively). External rotation at 90° abduction was 45° (60° passively). Internal rotation at 90° abduction was 50° (60° passively).

Supraspinatus testing elicited anterolateral pain and scaption weakness, with a positive Jobe test. Infraspinatus loading during external rotation revealed posterior pain with associated weakness. Bear Hug, Belly Press, and Lift Off tests were negative. Hornblower test was negative. The biceps tendon was markedly irritable within the groove, with a positive Speed test. The O'Brian test was negative.

Anterior instability testing provoked discomfort and demonstrated a positive apprehension–relocation–release triad. Posterior instability testing was unremarkable. The results of Kim and Jerk tests were negative. The acromioclavicular joint was stable and nontender, with a negative cross-body adduction test.

Constant-Murley score at presentation was 39%, comprising pain: 3.5/15; activities of daily living: 7.9/20; range of motion: 22/40; and strength: 6/25.

**Figure 3:**
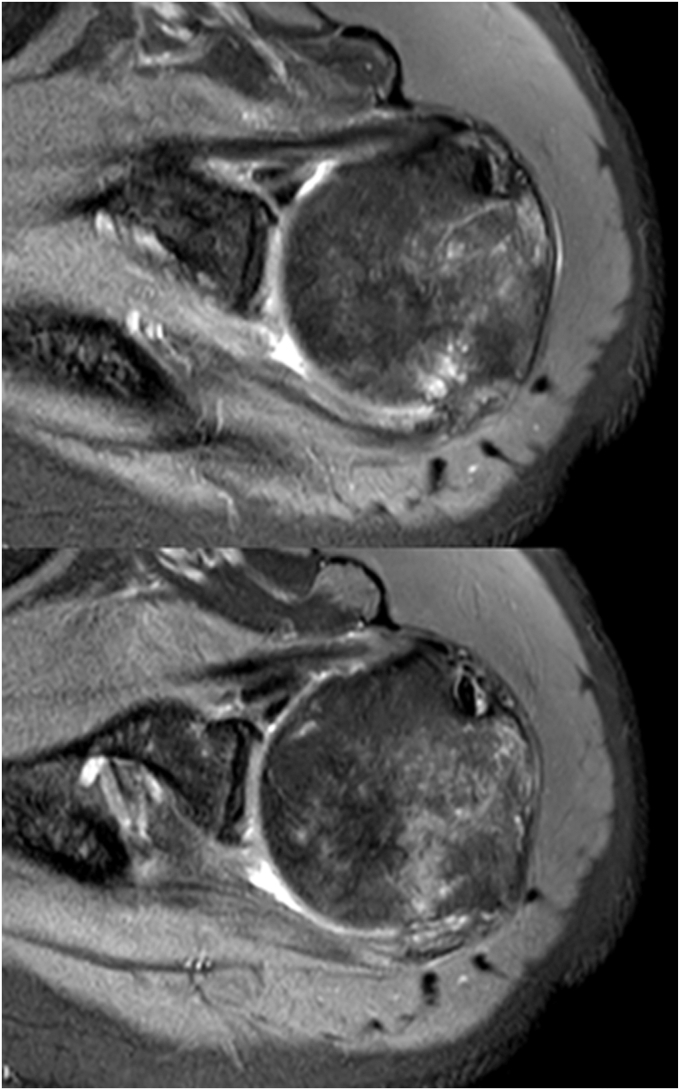
Magnetic resonance image performed preoperatively without intra-articular contrast (8 months and 9 days after the trauma).

**Figure 4:**
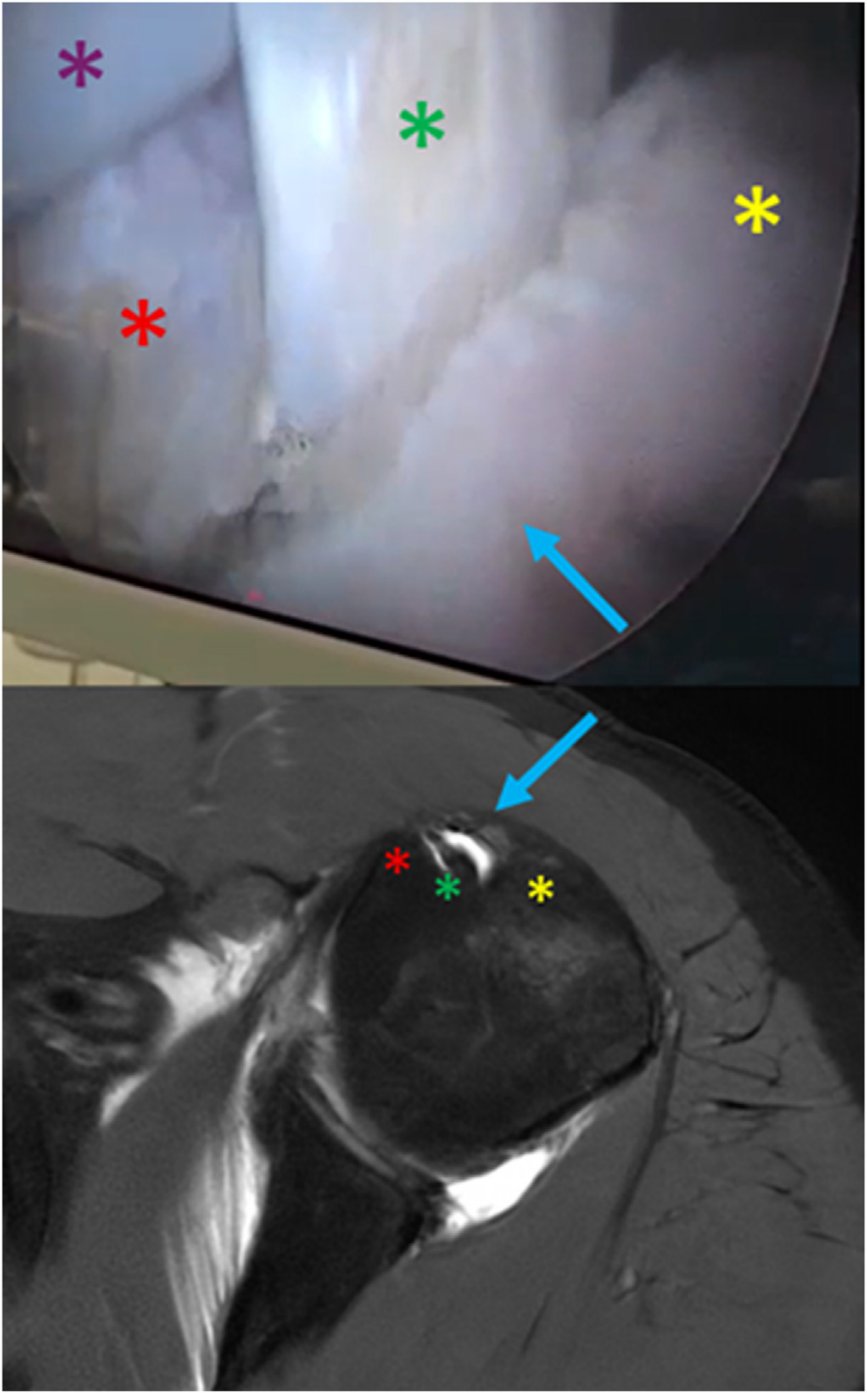
*Top*: Arthroscopic finding during surgery (9 months and 10 days after the polytrauma). *Bottom*: Magnetic resonance arthrogram performed preoperatively with intra-articular contrast (8 months and 23 days after the trauma). *∗*, *yellow*: greater tuberosity, *red*: lesser tuberosity, *green*: long head of biceps tendon, violet: electrocautery wand. *Blue arrow*: mature osseous bridge between the tuberosity.

Magnetic resonance arthrogram (MRA) with intra-articular gadolinium was performed on April 4, 2025. Both this contrasted image and the previous noncontrasted one (of March 21) revealed an articular-delaminated partial-thickness tear involving the supraspinatus and superior fibers of the infraspinatus tendons (posterosuperior cuff), with retraction and thickening of the articular layer consistent with a type 2a delaminated tear. Intrasubstance cleavage became indistinct due to chronicity and further retraction. The bursal layer appeared attenuated and weakened, with focal thinning. An associated anteroinferior labral tear and narrow-and-shallow on-track Hill-Sachs lesion were also noted. The anterior inferior glenohumeral ligament was irregular and thickened. Effusion was noted within the BG. Importantly, ossification of the THL was captured on both MRI and MRA but was overlooked and not recognized preoperatively (Figs. 3 and 4).

On April 22, 2025, the patient underwent arthroscopic labral and rotator cuff repair in the beach chair position. A type 2a delaminated tear of the rotator cuff and a labral tear extending from 3 o'clock to 6 o'clock position were confirmed. The intra-articular segment of the LHBT was severely swollen and inflamed, with longitudinal partial tearing and a superior labral anterior posterior grade two lesion. The capsule appeared hyperemic, and the rotator interval was thickened and contracted. Passive ER1 under anesthesia was limited to 45°.

Thorough rotator interval débridement was performed. A capsular release was achieved using a basket punch from the superior capsule to the upper margin of the anterior inferior glenohumeral ligament, with care taken to protect the subscapularis tendon and muscle. Postrelease, passive ER1 increased to 70°, passive external rotation at 90° abduction increased to 70°, and passive elevation to 170°. Using knotless anchors, the anterior labrum was repaired without capsular plication. The scope was then transitioned to the subacromial space, where subacromial bursectomy and decompression were completed.

During arthroscopic attempts to locate the LHBT for tenodesis, the tendon proved exceptionally difficult to identify. Using the exposed rotator interval window and subscapularis tendon as reference landmarks, lateral débridement uncovered the LT. Continued débridement along the leading edge of the bursal layer of supraspinatus enabled visualization of the intra-articular LHBT, which was subsequently tracked distally. The tendon was found to be encased within a well-organized lamellar cortical bony bridge spanning the LT and GT. The bridge formed a smooth, spherical osseous continuity between the tuberosities. Notably, there were no free-floating calcifications or tissue-laden deposits visualized or palpable on probing. Using the electric wand, the LHBT was mobilized and excursed repeatedly through the bone tunnel (Supplementary Video S1). The intratubercular segment of the LHBT did not exhibit the erythematous inflammation observed in its intra-articular segment; however, surface fraying and fatty degeneration, suggestive of chronic attritional changes, were evident.

Using a Kerrison rongeur, the bony bridge was resected piecemeal. The tactile feedback reported by the operating surgeon (M.J.) indicated the presence of hard, mature, and well-formed cortical bone. Direct extracorporeal assessment of the excised fragments also confirmed their organized, lamellar nature. Biceps tenodesis was completed in the lower BG using an onlay, single-anchor technique, followed by excision of the intertubercular and intra-articular segments of the LHBT. The posterosuperior cuff tear was then completed and repaired using a double-row configuration. Before concluding the procedure, anterior glenohumeral stability and the integrity of the tenodesis and cuff repair sites were verified under dynamic stress testing. The final passive range of motion was assessed and found to be stable and satisfactory.

Fragments of the bony bridge were not sent for histopathology evaluation due to financial limitations. The procedure was not formally recorded; however, a staff member was asked to record a video of the bridge on their phone, reflecting our unexpected encounter with this unprecedented pathology.

Postoperatively, the MRI and MRA studies, both acquired within the ninth month after trauma, were retrospectively scrutinized. The earlier MRI showed signs of incomplete ossification within the THL, whereas later MRA revealed complete THL ossification with frank synostosis of the tubercles. This serendipitous interval imaging appears to have captured a critical window in the post-traumatic progression of THL ossification. Notably, the patient did not receive pharmacologic intervention or any form of conservative treatment, including physical therapy, during the interval period between these imaging acquisitions. It's worth mentioning as well that the patient's pelvic and femoral shaft fractures did not get complicated by local or regional heterotopic ossification.

The patient was clinically assessed on July 9, 2025 (11 weeks postoperative). He was pain-free with no symptoms of instability. Anterior apprehension tests and provocative testing of supraspinatus and infraspinatus were negative. Biceps activation yielded no anterior shoulder pain.

## Discussion

This case illustrates the post-traumatic pathogenesis of THL ossification in a young nonarthritic shoulder. To our knowledge, this is the first report in literature documenting this lesion in a live human subject. Although radiologic evidence nine months after trauma demonstrated rapid progression from early to complete ossification over a 2-week interval, we refrain from attributing this transition precisely to the ninth post-traumatic month, recognizing the inherent limitations of magnetic resonance-based imaging in accurately characterizing mature osseous formation. The definite finding remains the complete ossification of the THL at nine months, confirmed through arthroscopic visualization, palpation, and resection.

We postulate that violent abduction and external rotation of the arm (Fig. 1) forced the LHBT medially against the LT and superiorly against THL, potentially inducing sudden, high-grade strain or even a tear of the THL. The extensive soft tissue disruption of the anterior shoulder likely triggered an exuberant regional inflammatory healing response (eg, adhesive capsulitis), potentiated by a systemic polytraumatic cytokine surge. We propose that exaggerated regenerative signaling triggered aberrant hyperplastic remodeling within the injured THL tissue, culminating in osseous metaplasia. Subsequent repetitive strain and compounded microtrauma absorbed by the healing THL during prolonged rehabilitation may have further catalyzed this metaplastic conversion, gradually replacing scar tissue with lamellar bone. Chronic remodeling consolidated into a mature cortical osseous bridge.

Wurnig^13^ retrospectively reviewed 641 patients who had undergone shoulder ultrasound and reported mineralization of the THL as a highly echogenic zone in four individuals (0.6%). However, no clinical correlation or further analysis using advanced imaging was provided. Complete ossification of the THL was first reported in 2013 by Singh,^11^ who incidentally identified the phenomenon in 1 of 100 cadaveric shoulder dissections. A second cadaveric case was described by Chidambaram et al^3^ in 2015, also observed in 1 of 100 specimens. The third report emerged in 2023 from Brazil, where Aragão et al^1^ discovered a similar ossified THL during routine dissection. To our knowledge, no other published cases of THL ossification in humans exist to date.

Interestingly, the only documented case of THL ossification in a live subject originates from veterinary literature. In 2022, Tilve et al^10^ described histologically confirmed osseous metaplasia of the THL in a Bengal cat presenting with lameness. Computed tomography revealed an osseous bridge enveloping the BG, and ultrasonography demonstrated a partial tear and inflammation of the bicipital tendon. Conservative treatment with intra-articular corticosteroids failed to yield sustained symptomatic relief. Surgical excision of the osseous bridge resulted in immediate resolution of lameness, with durable improvement noted at the 1-year follow-up. Histopathologic examination confirmed well-differentiated bone formation that replaced normal ligament collagen fibers, with a transition from fibrous dense tissue to a lamellar architecture that included osteoblasts, osteoclasts, and osteocytes and medullary cavities with blood vessels. This was classified as a well-differentiated osseous metaplasia of the THL. This finding is consistent with our observation that this process may represent true bone formation (osseous metaplasia) rather than native tissue mineralization.

Similar fibroblast-to-osteoblast transformation and lamellar bone production have been documented in various musculoskeletal lesions and must be distinguished from dystrophic calcium deposition within native tissue. True osseous metaplasia is well characterized in the spine, notably in the ossification of the posterior longitudinal ligament and in diffuse idiopathic skeletal hyperostosis.^7^ Post-traumatic ossification has been documented in the patellar tendon, the medial collateral ligament of the knee (Stieda-Pellegrini lesion), heterotopic ossification, and myositis ossificans traumatica.^4^^,^^6^ Importantly, this process is pathologically distinct from calcific tendonitis, which involves calcium deposition in a tissue that underwent fibrocartilaginous metaplasia (a different cellular lineage).^8^ Unlike osseous metaplasia, no true bone formation occurs in calcific tendonitis. Notably, traumatic injuries to the coracoacromial and coracoclavicular ligaments may trigger either calcium deposition or ossification, with the literature suggesting a roughly equal prevalence of each response.^2^ Perhaps this post-traumatic response represents a biological continuum—ranging from a limited dystrophic calcification to a complete lamellar bone formation. The threshold between these outcomes may hinge on local mechanical stress, aberrant chronic inflammation, and tissue-specific cytokine signaling.

## Disclaimers:

Funding: No funding was disclosed by the authors.

Conflicts of interest: The authors, their immediate families, and any research foundation with which they are affiliated have not received any financial payments or other benefits from any commercial entity related to the subject of this article.

Patient consent: Obtained.

## Supplementary data

Video 1

Theoretical Implications
