# Supplementary material for: Discovery of a fully ossified transverse humeral ligament during biceps tenodesis: a case report
Source: JSES Rev Rep Tech. 2025 Aug 29;5(4):1118–22. doi: 10.1016/j.xrrt.2025.08.011 (PMC12573477; doi:10.1016/j.xrrt.2025.08.011)
Supplement: Theoretical Implications [file mmc2.pdf]

Theoretical Implications of Transverse Humeral Ligament Ossification

These are ***theoretical***, ***plausible*** implications of the presence of an ossified transverse humeral ligament (THL) in the proximal humerus. Please note that there is ***no evidence*** to support these points,

1. **Disruption of Humeral Head Vascular Microcirculation**
   A rigid osseous bridge may compress or reroute peri-tuberosity microvasculature, potentially affecting humeral head perfusion and healing capacity after trauma or surgery.
2. **Potential for Neurological Irritation**
   Proximity of the ossified bridge to the anterior circumflex humeral artery and axillary nerve branches could theoretically contribute to neurovascular irritation or entrapment.
3. **Interference with Bone Marrow Signaling in Tuberosities**
   Ossification bridging the lesser and greater tuberosities may alter marrow-derived cytokine gradients, affecting local osteogenesis, tendon healing, or inflammatory modulation.
4. **Compromised Tendon Healing Environment**
   The ossified tunnel may create a hypovascular or a mechanical hostile environment for the LHBT, impairing healing after tenodesis or conservative management of tendinopathy.
5. **Impact on Tendon-to-Bone Healing Dynamics**
   In repairs involving the supraspinatus or subscapularis, local ossification may skew native load distribution, delaying enthesis maturation.
6. **Altered Biceps Pulley Mechanics**
   Ossification may disrupt the coordinated function of the biceps pulley system, including the superior glenohumeral and coracohumeral ligaments, leading to instability or aberrant LHBT tracking.
7. **Reduced Tendon Excursion During Motion**
   A non-expendable and non-malleable osseous tunnel may limit the natural excursion of the LHBT during shoulder flexion and rotation, potentially contributing to pain or mechanical restriction.
8. **Attritional/ Stenosing Pathology of LHBT:**
   Encasement within a rigid osseous tunnel may lead to frictional wear, fiber degeneration, and mechanical stenosis of the LHBT over time – an entrapment variant of LHBT tendinopathy distinct from classic tenosynovitis or instability.
9. **Altered Glenohumeral Kinematics**
   A rigid osseous bridge across the tuberosities may restrict subtle gliding motions of the humeral head, potentially contributing to micro-instability or altered joint mechanics over time.
10. **Influence on Osteoarthritic Progression Modeling**
    Static anterior anchors like ossified THL may alter joint kinetics and cartilaginous shear forces, offering insight into the pathway from instability to arthritis.
11. **Role in Altering Glenohumeral Contact Pressures**
    Rigid tethering across tuberosities may elevate local pressure zones during overhead loading—useful data for implant design and motion capture studies.

12. **Influence on Shoulder Kinetics During Sleep or Resting Postures**
A rigid anterior bridge may alter passive shoulder positioning during sleep, potentially contributing to nocturnal pain or positional neuropathies.
13. **Compromised Subdeltoid Space Dynamics**
The bony bridge may reduce subdeltoid compliance, affecting gliding of the rotator cuff and contributing to stiffness or impingement-like symptoms.
14. **Coracoid Impingement Potential:**
A prominent ossified bridge may encroach on the subcoracoid space, predisposing to mechanical impingement between the newly formed cortical structure and the coracoid process.
15. **Impact on Shoulder Proprioception**
Encasement of the LHBT and altered ligamentous architecture may impair mechanoreceptor feedback, subtly affecting joint position sense and neuromuscular control.
16. **Impact on Shoulder Biomechanics in Overhead Athletes**
In high-demand shoulders, especially in throwing athletes, ossified THL may alter force transmission across the rotator cuff and biceps pulley system, increasing susceptibility to overuse injuries.
17. **Potential Role in Post-Traumatic Shoulder Stiffness**
THL ossification may contribute to capsular tethering or anterior interval rigidity, exacerbating adhesive capsulitis-like presentations in post-trauma patients.
18. **Altered Stress Distribution Across the Rotator Cuff Footprint**
The ossified THL may act as a stress riser or redistribute tensile loads across the supraspinatus and infraspinatus insertions, possibly influencing tear propagation or repair integrity.
19. **Reduced Efficacy of Imaging-Guided Interventions**
Ossified THL may obscure the bicipital groove on ultrasound or MRI, complicating diagnostic injections, aspirations, or guided procedures targeting the LHBT or adjacent structures.
20. **Barrier to Endoscopic or Minimally Invasive Techniques**
In cases where arthroscopic access to the groove is required, ossified THL may limit visualization or instrumentation, necessitating conversion to open procedures.
21. **Anomalous Landmarks During Diagnostic Arthroscopy:**
THL ossification may distort familiar anatomic cues in the rotator interval, complicating orientation for procedures such as SLAP repair or subscapularis augmentation.
22. **Tactile Resistance During Instrumentation:**
Ossified THL may provide unexpected feedback during scope insertion or debridement, risking misdirection or collateral damage without careful intra-op palpation.
23. **Need for Accessory Portals or Extended Dissection:**
Standard arthroscopic portals may not suffice; anterior interval scarring or ossification may necessitate open conversion or novel access angles.
24. **Instrument Navigation Hazards:**
Suture passing or knot tying instruments may deflect unpredictably around the ossified bridge, increasing risk of iatrogenic cuff abrasion.
25. **Risk of Iatrogenic Injury During Surgery**
Unexpected ossification may increase the risk of damaging adjacent structures (e.g.,

subscapularis, neurovascular bundle) during arthroscopy or open procedures due to altered anatomy.

26. **Proximal Humerus Reconstruction Complexity:**
    A bony bridge spanning the tuberosities may obscure anatomical landmarks and distort humeral contour, complicating open reduction or fixation strategies.
27. **Anatomic Shoulder Arthroplasty Challenges:**
    Ossified THL may impair humeral orientation by altering visual or tactile cues critical for anatomic alignment, especially during press-fit stem placement. Humeral component retroversion may be affected.
28. **Biceps Tenodesis Difficulty:**
    Complete ossification across the bicipital groove can obscure or distort the LHBT trajectory, rendering standard groove-based tenodesis technically demanding or unfeasible.
29. **Challenge in Revision Surgery or Hardware Placement**
    In cases requiring revision tenodesis or proximal humerus hardware, ossified THL may obstruct anchor placement or necessitate additional bone work.
30. **Influence on Postoperative Rehabilitation Outcomes**
    Altered biomechanics and tendon containment may affect responsiveness to physical therapy, especially in protocols relying on LHBT mobility or groove-based orientation.
31. **Misleading Radiologic Interpretation**
    THL ossification may mimic pathological calcifications or neoplastic lesions on imaging, leading to diagnostic confusion or unnecessary workup.
32. **Pseudo-Neoplastic Presentation:**
    Dense ossification with cortical margins could radiographically mimic osteoid osteoma or heterotopic bone tumors, triggering unnecessary alarm or biopsy.
33. **Risk of Misdiagnosis:**
    An ossified THL may be misdiagnosed as accessory ossicles, subchondral cysts, or calcific tendinopathy, delaying treatment.
34. **Risk of Misidentification as a Congenital Anomaly**
    In younger patients or those without trauma history, ossified THL may be misinterpreted as a congenital synostosis or developmental variant, delaying appropriate management.
35. **Potential to Serve as a Radiologic Marker of Prior High-Energy Trauma**
    THL ossification may retrospectively signal prior dislocation or capsular disruption, offering forensic or diagnostic value in unclear trauma histories.
36. **Potential for Misclassification in Imaging-Based Scoring Systems**
    Ossified THL may be misinterpreted as calcific tendinopathy or tuberosity hypertrophy, skewing radiologic grading systems used for cuff pathology or arthroplasty planning.
37. **Complication in preoperative planning software**
    The presence of an ossified THL may distort automatic segmentation and computer measurement of posterior humeral head subluxation
38. **Complication in Imaging-Based Navigation Systems**
    Unexpected ossification may distort anatomical landmarks used in robotic or computer-assisted shoulder surgery, reducing accuracy of implant placement or anchor targeting.
